# A microRNA‐clinical prognosis model to predict the overall survival for kidney renal clear cell carcinoma

**DOI:** 10.1002/cam4.4148

**Published:** 2021-07-21

**Authors:** Yating Zhan, Rongrong Zhang, Chunxue Li, Xuantong Xu, Kai Zhu, Zhan Yang, Jianjian Zheng, Yong Guo

**Affiliations:** ^1^ Key Laboratory of Diagnosis and Treatment of Severe Hepato‐Pancreatic Diseases of Zhejiang Province The First Affiliated Hospital of Wenzhou Medical University Wenzhou China; ^2^ Department of Urology The First Affiliated Hospital of Wenzhou Medical University Wenzhou China

**Keywords:** kidney renal clear cell carcinoma, microRNA, nomogram, prognosis model

## Abstract

Numerous studies have shown that microRNA (miRNA) serves as key regulatory factors in the origin and development of cancers. However, the biological mechanisms of miRNAs in kidney renal clear cell carcinoma (KIRC) are still unknown. It is necessary to construct an effective miRNA‐clinical model to predict the prognosis of KIRC. In this study, 94 differentially expressed miRNAs were found between para‐tumor and tumor tissues based on the Cancer Genome Atlas (TCGA) database. Seven miRNAs (hsa‐miR‐21‐5p, hsa‐miR‐3613‐5p, hsa‐miR‐144‐5p, hsa‐miR‐376a‐5p, hsa‐miR‐5588‐3p, hsa‐miR‐1269a, and hsa‐miR‐137‐3p) were selected as prognostic indicators. According to their cox coefficient, a risk score formula was constructed. Patients with risk scores were divided into high‐ and low‐risk groups based on the median score. Kaplan–Meier curves analysis showed that the low‐risk group had a better survival probability compared to the high‐risk group. The area under the ROC curve (AUC) value of the miRNA model was 0.744. In comparison with clinical features, the miRNA model risk score was considered as an independent prognosis factor in multivariate Cox regression analysis. In addition, we built a nomogram including age, metastasis, and miRNA prognostic model based on the results of multivariate Cox regression analysis. The decision curve analysis (DCA) revealed the clinical net benefit of the prognostic model. Gene set enrichment analysis (GSEA) results suggested that several important pathways may be the potential pathways for KIRC. The results of Gene Ontology (GO) and Kyoto Encyclopedia of Genes and Genomes (KEGG) enrichment analysis for the target genes of 7 miRNAs revealed that miRNAs may participate in KIRC progression via many specific pathways. Additionally, the levels of seven prognostic miRNAs showed a significant difference between KIRC tissues and adjacent non‐tumorous tissues. In conclusion, the miRNA‐clinical model provides an effective and accurate way to predict the prognosis of KIRC.

## Graphical abstract

In this study, we constructed a novel 7‐miRNA risk model for KIRC, which may be an independent predictor for KIRC. Moreover, a nomogram including the above risk model and clinical features could provide a better way to evaluate the prognosis of KIRC patients.

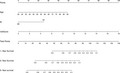

## 1 INTRODUCTION

Renal cell carcinoma (RCC) is known as one of the most common cancers throughout the world. Generally, RCC is divided into three types: kidney renal clear cell carcinoma (KIRC), kidney renal papillary cell carcinoma (KIRP), and malignancies of the chromophobe.^1^ Among them, KIRC is the most common type of kidney cell cancers and accounts for approximately 80–90% of RCC.^2^, ^3^ In most cases, KIRC is resistant to radiotherapy and chemotherapy, and the main treatment is surgery.^4^ Despite early surgical treatment is taken, 30% of patients are at a relatively higher risk of developing metastasis and recurrence.^5^ Therefore, KIRC still threats human health and life as a malignant disease. Until now, the roles of microRNAs (miRNAs) in KIRC are still largely unknown and few crucial biomarkers have been found. Therefore, it is necessary to explore effective biomarkers for the prognosis of KIRC.

MiRNAs, consisting of 21–23 nucleotides, are evolutionarily conserved single‐strand endogenous noncoded RNAs that widely exist in humans and animals.^6^ MiRNAs are generally not translated into proteins, but they are closely associated with the complex cell mechanisms responsible for gene expression. In addition, miRNAs play a crucial role in various human cancers.^7^ For example, miR‐21 could influence the invasiveness and angiogenesis of renal cell carcinoma cells by the PDCD4/c‐Jun (AP‐1) signaling pathway.^8^ Feng et al. revealed that circRNA_001287 promotes the proliferation of renal cell carcinoma through miR‐144/CEP55 signaling pathway.^9^

In this study, we aimed to construct a novel prognostic model including miRNA signature and clinical factors. Mature miRNA sequencing data and the corresponding clinical information of KIRC were downloaded from the Cancer Genome Atlas (TCGA) database. Then, seven miRNAs were found as important prognosis indicators of KIRC by Cox regression analysis and Lasso analysis. Subsequently, we established a seven miRNAs‐clinical model to predict the prognosis of KIRC patients. The expressions of the 7 miRNAs between KIRC tissues and adjacent non‐tumorous tissues were also examined.

## 2 MATERIALS AND METHODS

### 2.1 Data Processing

We downloaded the mature miRNA sequencing data of KIRC from the TCGA database, including 71 normal and 545 tumor tissues. Besides, the clinical information of KIRC patients was obtained. The patients with the survival time of 0 day were excluded. The clinical information table is shown in Table 1. In addition, the mRNA expression data were downloaded. The edgeR R package was employed to perform analysis to identify differentially expressed miRNAs (DEmiRNAs) and differentially expressed mRNAs (DEmRNAs).^10^ The DEmiRNAs and DEmRNAs were selected with the conditions: | log fold change (FC) | >1.5 and adjusted p value <0.05.

**TABLE 1:** Clinical characteristics of patients with KIRC from the TCGA database

Variables	TCGA set, n = 512
Death(%)	170 (33.20%)
Age(years)	57 ± 31
Gender(%)	
Female	178 (34.77%)
Male	334 (65.23%)
Grade(%)	
G1	12 (2.34%)
G2	217 (42.38%)
G3	201 (39.26%)
G4	74 (14.45%)
Missing	8 (1.56%)
Stage(%)	
Stage I	249 (48.63%)
Stage II	55 (10.74%)
Stage III	123 (24.02%)
Stage IV	82 (16.02%)
Missing	3 (0.59%)
T(%)	
T1	255 (49.80%)
T2	67 (13.09%)
T3	179 (34.96%)
T4	11 (2.15%)
M(%)	
M0	404 (78.91%)
M1	78 (15.23%)
Missing	30 (5.86%)
N(%)	
N0	227 (44.34%)
N1	17 (3.32%)
Missing	268 (52.34%)

### 2.2 miRNA model construction

Univariate Cox regression analysis was performed by using the survival R package and 24 DEmiRNAs related to prognosis were selected by setting *p* < 0.01. Lasso Cox regression was performed to remove miRNAs that were highly overfitted.^11^ Therefore, 14 miRNAs were chosen by LASSO Cox regression analysis. Finally, seven miRNAs were selected by multivariate Cox regression analysis and a risk score formula was constructed according to their cox coefficient. The formula was as follows: Risk score = ∑Coef miRNAs * log2 (Expression of miRNAs +1). Then, each patient's risk score was calculated based on the formula and all patients were divided into high‐ and low‐ risk groups according to the median score. In addition, we used the receiver operating characteristic (ROC) curve to evaluate the specificity and sensitivity of the prognostic miRNAs risk model by survivalROC R package.

### 2.3 Functional enrichment analysis

In order to better understand the functional enrichment pathways of high‐ and low‐risk groups, we performed gene set enrichment analysis (GSEA). The important pathways were picked out by NOM‐*p* value < 0.05. Then, we predicted the target genes of these 7 miRNAs by website miRWalk and took the intersection with DEmRNAs.^12^ Target DEmRNAs were submitted to the website DAVID to perform Gene Ontology (GO) and Kyoto Encyclopedia of Genes and Genomes (KEGG) enrichment analysis. The terms with *p* less than 0.05 were considered statistically significant.

### 2.4 Clinical independence prognosis analysis for the risk model

To evaluate the independent prognostic ability of the risk model, both clinical features (age, gender, grade, stage, T, M, and N) and the 7‐miRNA risk model were analyzed into the univariate and multivariate Cox regression analyses. The factors meeting *p* < 0.05 were considered statistically significant. The results of multivariate Cox analysis with *p* < 0.05 were considered as independent prognostic factors to predict the prognosis of KIRC.

### 2.5 Construction of a miRNAs‐clinical nomogram

The independent prognostic factors of KIRC were included in the miRNAs‐clinical nomogram.^13^ The calibration curve analysis was utilized to evaluate the accuracy between the predicted and actual mortalities. The ROC and decision curve analysis (DCA) for the age, M, miRNA, and combined models (age, M, and miRNA model) were simultaneously analyzed.^14^ The clinical net benefit of the established nomogram was shown in the DCA.

### 2.6 7 miRNA expression levels between paired KIRC and adjacent non‐tumorous tissues

A total of 20 paired KIRC and adjacent non‐tumorous tissue samples were obtained from the First Affiliated Hospital of Wenzhou Medical University. The Ethics Committee of the First Affiliated Hospital of Wenzhou Medical University approved the use of clinical samples. The patients/participants provided their written informed consent to participate in this study. The expressions of miRNAs were measured by quantitative real‐time PCR (qRT‐PCR). The total RNA content of KIRC and adjacent non‐tumorous tissue was extracted by TRIzol reagent. Then, miRNA was reverse transcribed into cDNA using ribo SCRIPTTM Reverse Transcription Kit with the volume of 10 μL. The expression level of miRNA was calibrated by U6. Real‐time PCR was performed by using SYBR Green Master Mix on the 7500 Fast quantitative PCR System (Applied Biosystems). The Ct value of each well was recorded and the relative quantification of the product was performed by using the 2^‐ΔCt^ method. Differences between para‐tumor and KIRC group were compared using a Student's *t*‐test. *p*‐value < 0.05 was considered statistically significant.

## 3 RESULTS

### 3.1 Identification of DEmiRNAs and DEmRNAs in KIRC

**FIGURE 1:**
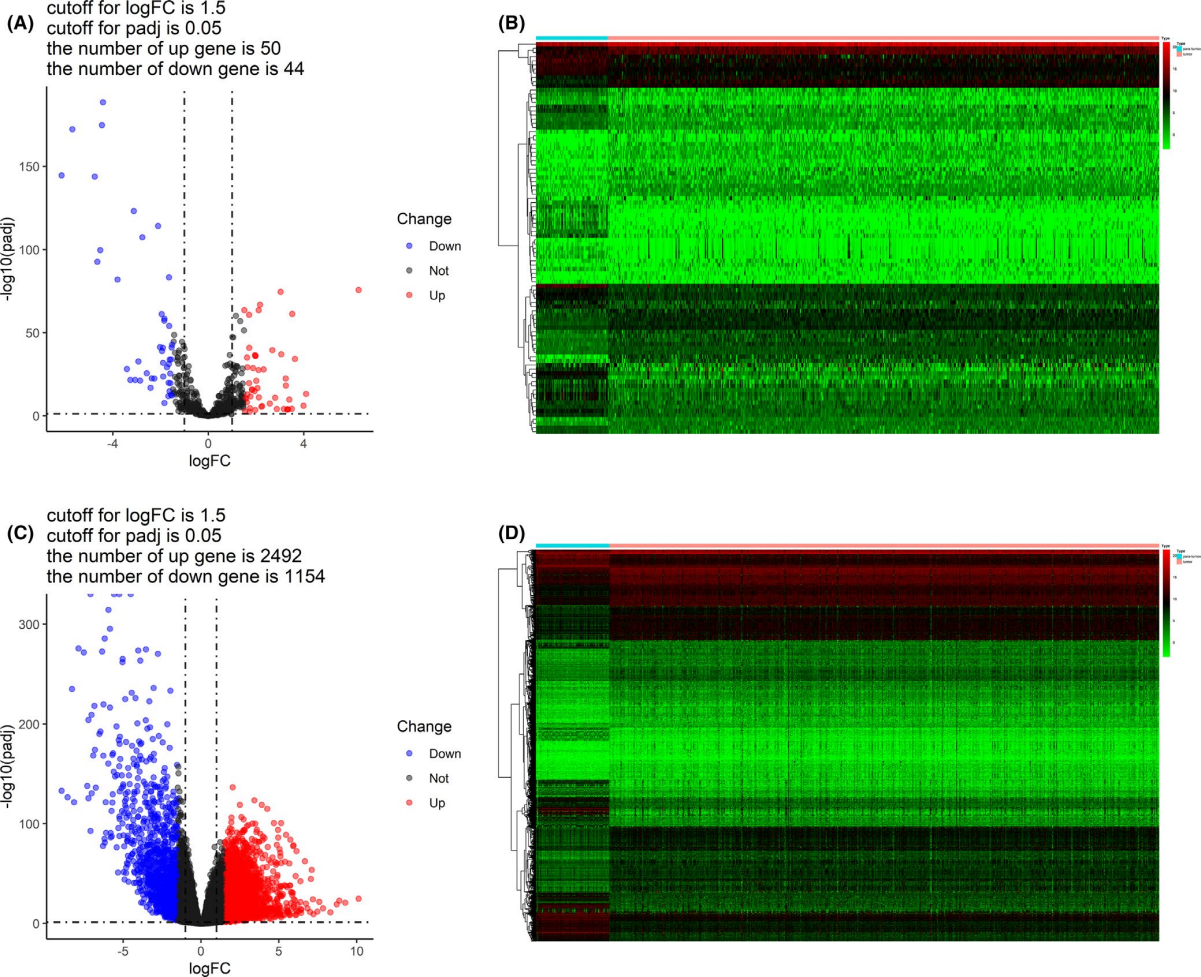
Differentially expressed miRNAs and mRNAs. (A) Volcano plot of differentially expressed miRNAs. (B) Heat map of differentially expressed miRNAs. (C) Volcano plot of differentially expressed mRNAs. (D) Heat map of differentially expressed mRNAs

Using the edgeR R package, we identified the DEmiRNAs and DEmRNAs between KIRC and para‐tumor kidney tissues from the TCGA database, with the adjusted *p* < 0.05 and |logFC|>1.5 as the thresholds. There were 94 DEmiRNAs (50 up and 44 downregulated) and 3646 DEmRNAs (2492 up and 1154 downregulated) in KIRC. The distributions of the DEmiRNAs and DEmRNAs were shown in the volcano plots (Figure 1A,C). In addition, the expression profiles of DEmiRNAs and DEmRNAs in KIRC were shown in the heat maps (Figure 1B,D).

### 3.2 Identification and selection of significant miRNAs

Next, we investigated the correlation between DEmiRNAs and KIRC survival, and 4 patients with the survival time of 0 day were excluded from 516 patients. Univariate Cox regression analysis was performed in 94 DEmiRNAs. Then, a total of 24 prognostic‐related miRNAs were found (*p* < 0.01, Table S1). Moreover, the Lasso Cox regression was applied to punish every variable to screen variables. Fourteen miRNAs were picked out by Lasso according to the minimal λ value (Figure S1). Finally, seven significant KIRC prognosis‐related miRNAs (hsa‐miR‐21‐5p, hsa‐miR‐3613‐5p, hsa‐miR‐144‐5p, hsa‐miR‐376a‐5p, hsa‐miR‐5588‐3p, hsa‐miR‐1269a, and hsa‐miR‐137‐3p) were chosen by the multivariate Cox regression analysis. We next constructed a risk score formula based on the expression and cox coefficient of the seven miRNAs to predict the prognosis of KIRC:

Risk score = (0.4998*hsa‐miR‐21‐5p) + (0.5166*hsa‐miR‐3613‐5p) + (−0.2193*hsa‐miR‐144‐5p) + (0.1695*hsa‐miR‐376a‐5p) + (−0.1451*hsa‐miR‐5588‐3p) + (0.0550*hsa‐miR‐1269a) + (0.1615*hsa‐miR‐137‐3p).

**FIGURE 2:**
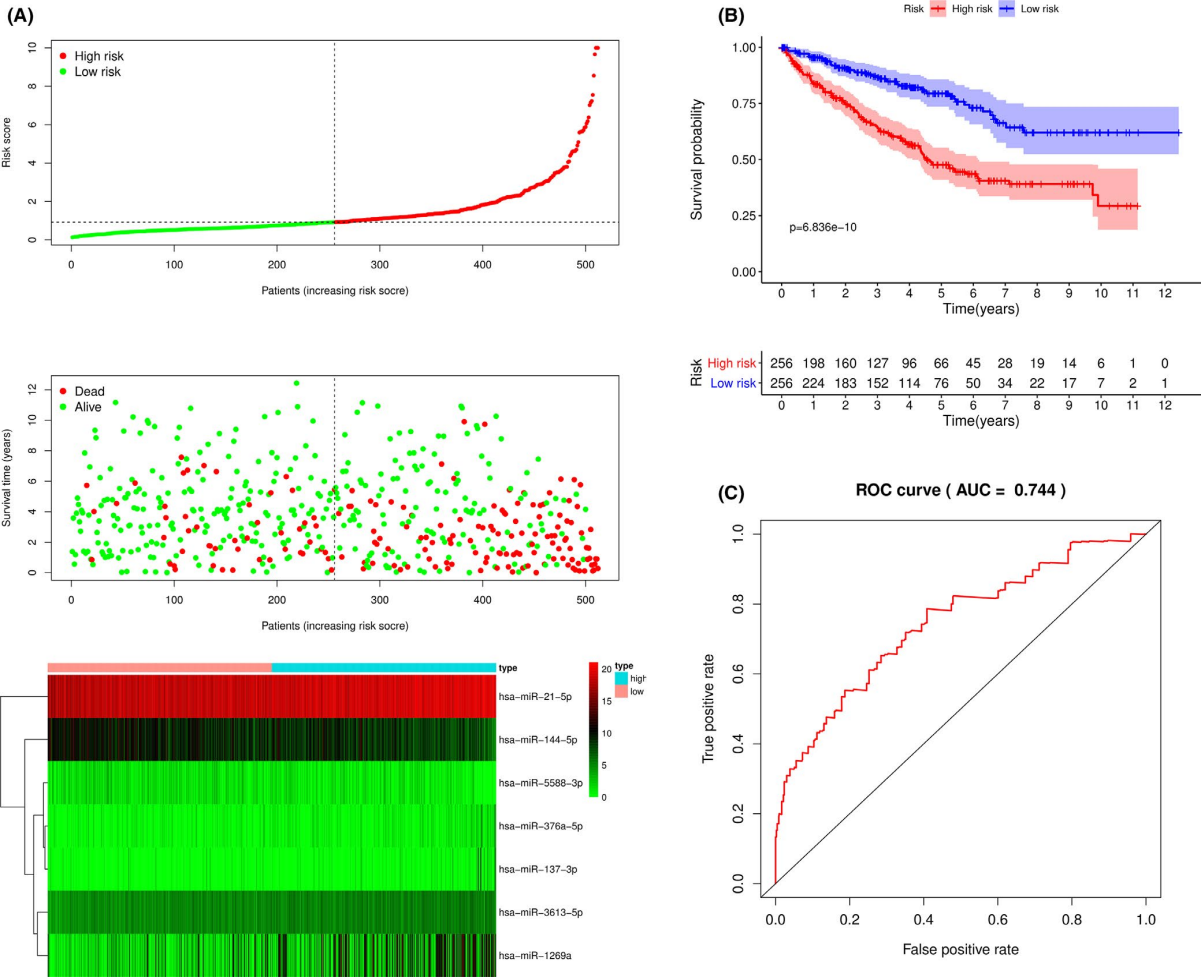
The 7‐miRNA signature associated with overall survival of KIRC. (A) The upper panel represents risk score distribution for each patient, the middle panel shows the patient distribution with increasing risk value, and the lower panel represents the expressions of 7 prognostic miRNAs. (B) Kaplan‐Meier curve analysis for the patients in KIRC between the high‐ and low‐risk group. (C) ROC curve analysis for the 7‐miRNA model for 1 year

**FIGURE 3:**
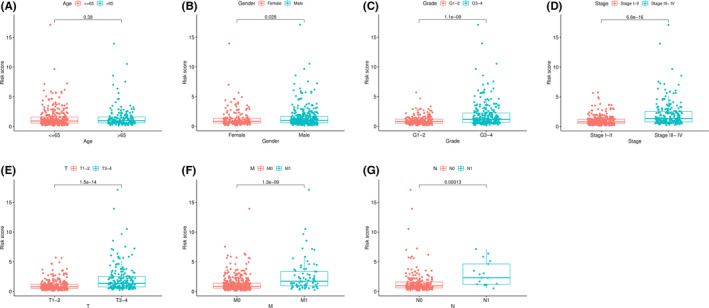
The correlation between risk scores and clinical features. Distribution of risk scores stratified by age (A), gender (B), grade (C), stage (D), T (E), M (F), and N (G)

In this formula, the expressions of miRNAs were taken the log2 value. Then, the risk scores of each patient were calculated by the risk formula. The patients were divided into high‐ and low‐risk groups according to the median score. The results of the risk score distribution, survival status, and miRNA expression showed that patients in the high‐risk group had a higher probability of death than those with low risk (Figure 2A). The Kaplan–Meier curve indicated that patients in the low‐risk group had better outcomes than those in the high‐risk group (*p *< 0.01) (Figure 2B). The 1‐, 3‐, and 5‐year survival probability of the high‐risk group was 84.0%, 64.4%, and 47.6%. The 1‐, 3‐, and 5‐year survival probability of the low‐risk group was 95.5%, 86.6%, and 79.4%. Our data suggest that patients in the low‐risk group have a longer survival time. Moreover, the ROC curve was used to evaluate the accuracy of the 7‐miRNA model (Figure 2C). Notably, the area under the ROC curve (AUC) of the above model was 0.744. Besides, we examined the relation between risk scores and clinical features. There was no relation between age and risk scores (Figure 3A). Compared with female patients, higher risk scores were found in male patients (Figure 3B). The risk scores were increased along with higher level of grade, stage T, M, and N (Figure 3C–G). GSEA analysis was performed to explore the potential pathways between the high‐ and low‐risk groups. The results of GSEA indicated that several pathways including the DNA replication, P53 signaling pathway, adipocytokine signaling pathway, fatty acid metabolism, mTOR signaling pathway, PPAR signaling pathway, ErbB signaling pathway, and insulin signaling pathway, may be involved in the progression of KIRC (Figure S2).

### 3.3 GO and KEGG enrichment analysis for 7‐miRNA target genes

**FIGURE 4:**
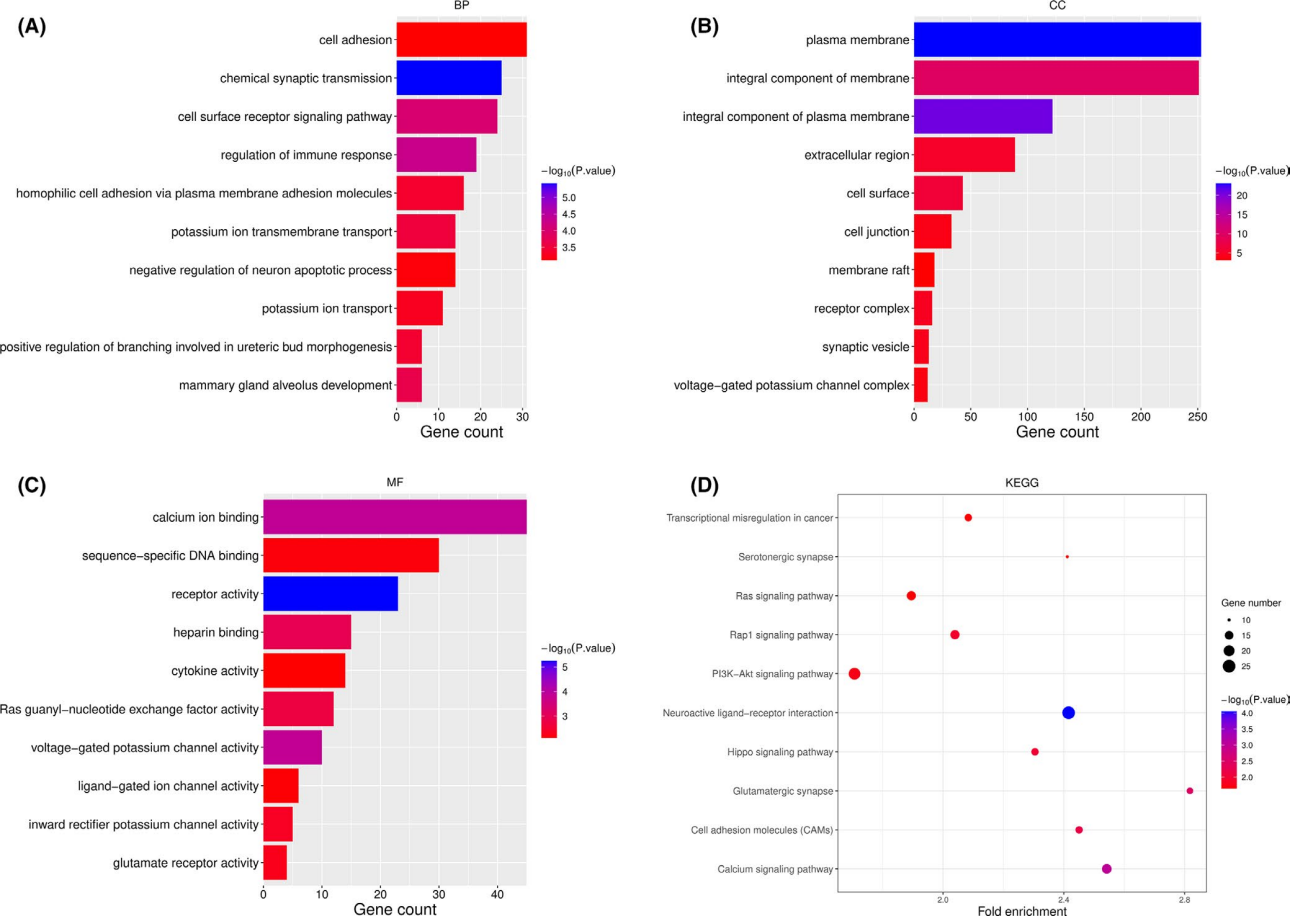
GO and KEGG enrichment analysis of target genes for 7 significant miRNAs. (A) Biological Process (BP). (B) Cellular Component (CC). (C) Molecular Function (MF). (D) KEGG

To explore the downstream mechanisms of the seven miRNAs, the potential target genes were predicted via miRWalk. Then, we set the binding score as 1 and took the intersection of predicted genes with DEmRNAs. Subsequently, 675 target genes were selected. To determine the roles of the 7‐miRNA in KIRC, we analyzed the enrichment functions of these selecting target genes by DAVID. We visualized the top 10 terms under the condition of *p*<0.05. It was found that biological process (BP), the terms of cell adhesion, cell surface receptor signaling pathway, regulation of immune response, potassium ion transmembrane transport, potassium ion transport, and positive regulation of branching involved in ureteric bud morphogenesis may be associated with KIRC progression (Figure 4A). As indicated by Figure 4B, the cellular component (CC) of target genes was significantly enriched in both plasma membrane and ion channel of renal cells surface. In addition, the molecular function (MF) of the target genes is correlated with calcium ion binding, voltage‐gated potassium channel activity, voltage‐gated potassium channel activity, and inward rectifier potassium channel activity (Figure 4C). The above target genes were also found to be involved in transcriptional misregulation in cancer, Ras signaling pathway, Rap1 signaling pathway, PI3K‐Akt signaling pathway, Hippo signaling pathway, and calcium signaling pathway pathways, as shown by KEGG results (Figure 4D).

### 3.4 Independent prognostic role of the miRNA signature

**FIGURE 5:**
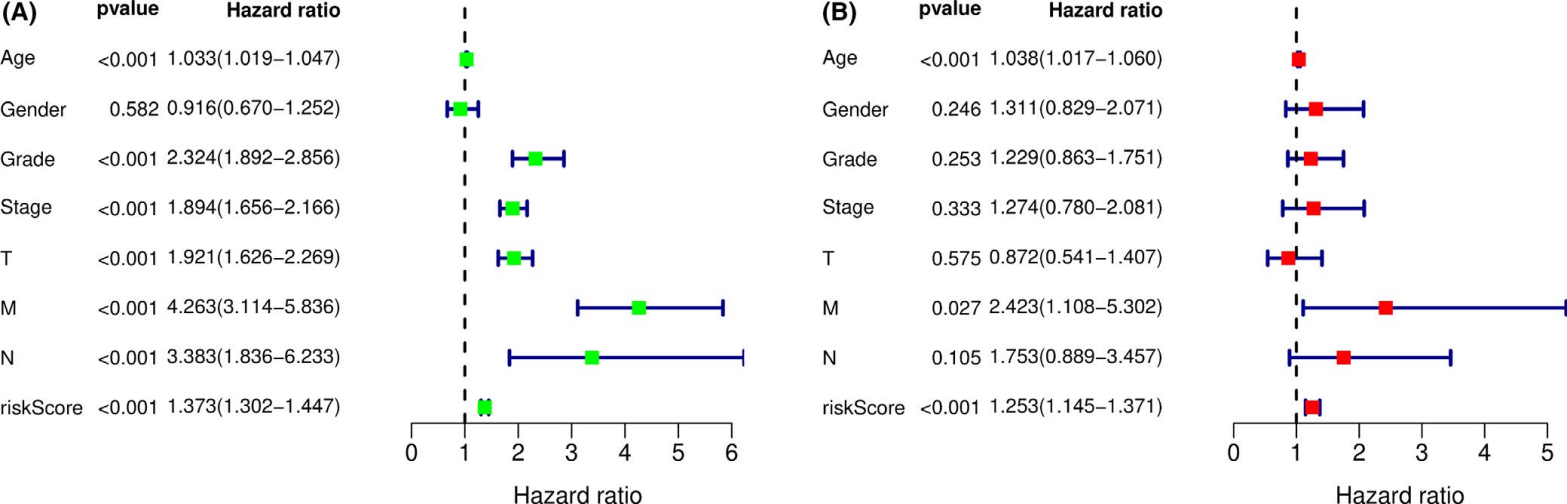
Independent prognosis analysis between clinical features and 7‐miRNA prognostic model. (A) Univariate Cox regression analysis between clinical features and 7‐miRNA prognostic risk score. (B) Multivariate Cox regression analysis between clinical features and 7‐miRNA prognostic risk score

Both univariate and multivariate Cox regression analyses were performed to explore the independent prognostic ability of the 7‐miRNA model with the clinical information including age, gender, grade, stage, T, M, and N. Univariate Cox analysis revealed that age (*p *< 0.001), grade (*p *< 0.001), stage (*p *< 0.001), T (*p *< 0.001), M (*p *< 0.001), N (*p *< 0.001), and miRNAs model risk score (*p *< 0.001) were significant risk factors associated with the prognosis of KIRC (Figure 5A). Multivariate Cox regression analysis demonstrated that age (*p *< 0.001), M (*p *= 0.027), and miRNA signature risk score (*p*<0.001) may be the independent prognostic factors (Figure 5B).

### 3.5 Stratified survival analysis

**FIGURE 6:**
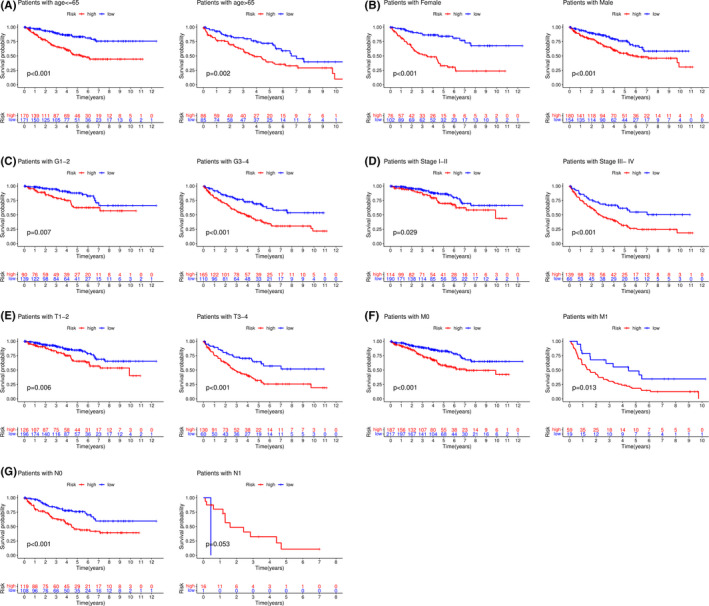
Survival analysis of all high‐ and low‐risk group patients stratified by clinical features. Kaplan‐Meier curves for patients in different level groups of age (A), gender (B), grade (C), stage (D), T (E), M (F), and N (G)

We also explored the accuracy of survival prediction in both high‐ and low‐risk groups with clinical features. As shown in Figure 6, patients with low risk had a better survival probability than those with high risk in most clinical sub‐groups. However, no significant results were found in the N1 sub‐group due to the relatively low sample size.

### 3.6 A nomogram with 7‐miRNA model and clinical features

**FIGURE 7:**
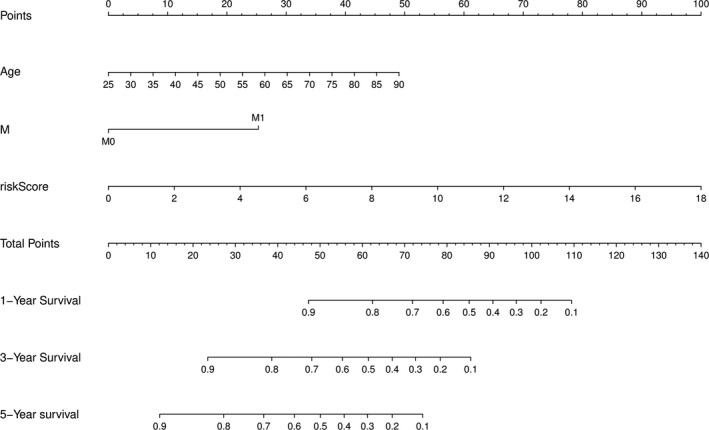
The miRNA‐clinical nomogram to predict the survival probability of KIRC

**FIGURE 8:**
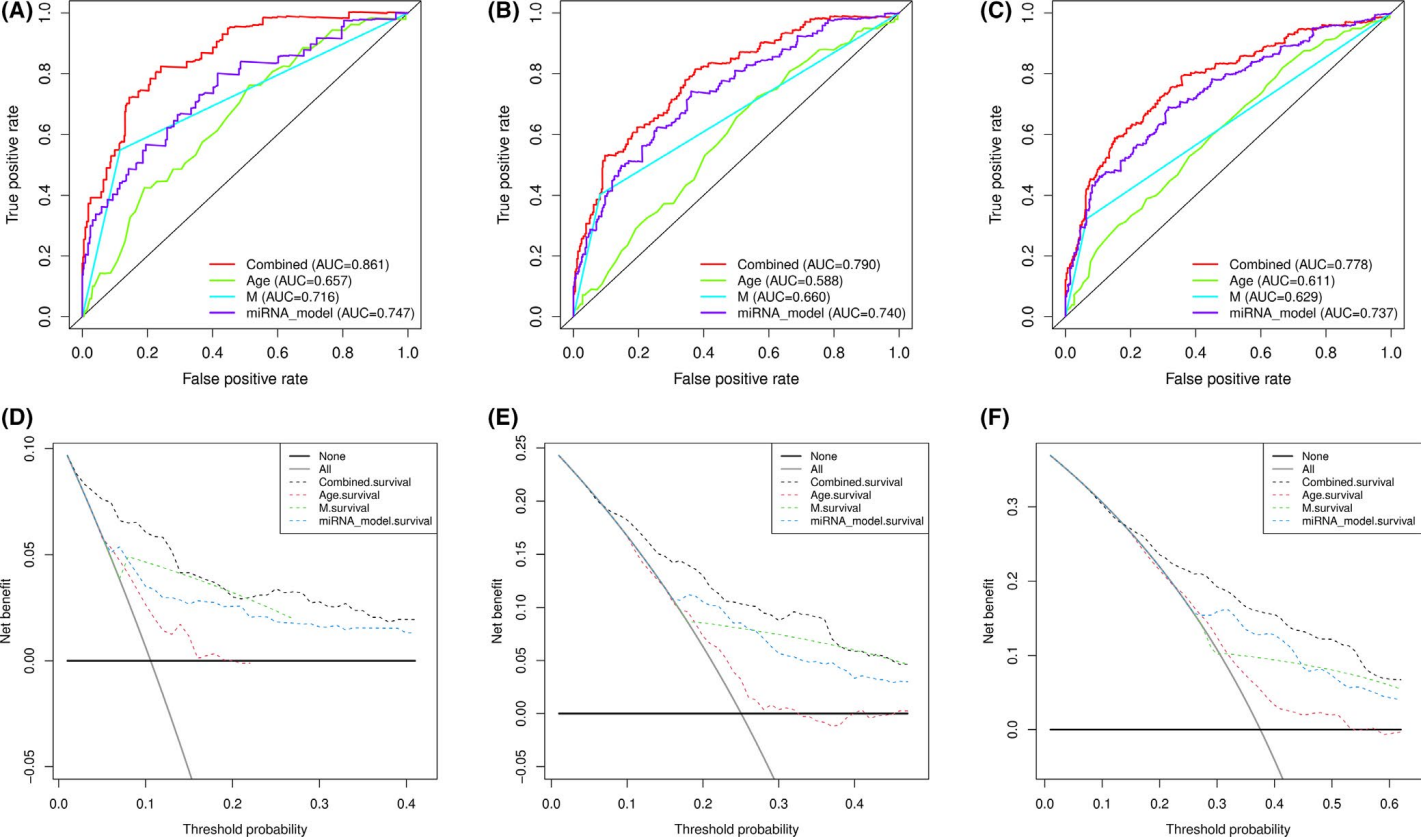
The ROC curve and DCA curve analysis to evaluate the accuracy of the nomogram at 1, 3 and 5 years, respectively. (A–C) ROC curve analysis of the nomogram compared for 1, 3, and 5 years. (D–F) DCA curve analysis of the nomogram compared for 1, 3, and 5 years

To determine the evaluation of miRNAs prognostic model and clinical features in the prognosis of KIRC patients, we built a nomogram that combined the miRNAs prognostic model with the clinical features such as age and metastasis (Figure 7). Moreover, the calibration plots of this nomogram for 1, 3, and 5 years are shown in Figure S3A–C. All the blue full lines were near to the broken line, suggesting that the combined model had a good performance in predicting the prognosis of KIRC. The AUC values for age, metastasis, and miRNAs prognostic model were additionally evaluated. As shown in Figure 8A–C, the AUC values for 1‐, 3‐, and 5‐year overall survival (OS) were 0.657, 0.588, and 0.611 in age, 0.716, 0.660, and 0.629 in metastasis, and 0.747, 0.740, and 0.737 in the miRNAs prognostic model, respectively. Interestingly, the AUC values were obviously increased in the combined model. Furthermore, DCA was used to evaluate the efficiency of this nomogram. As shown in Figure 8D–F, our miRNAs model with clinical factors showed a better net benefit in predicting OS.

### 3.7 Verification of seven miRNAs expression levels between paired KIRC and adjacent non‐tumorous tissues

**FIGURE 9:**
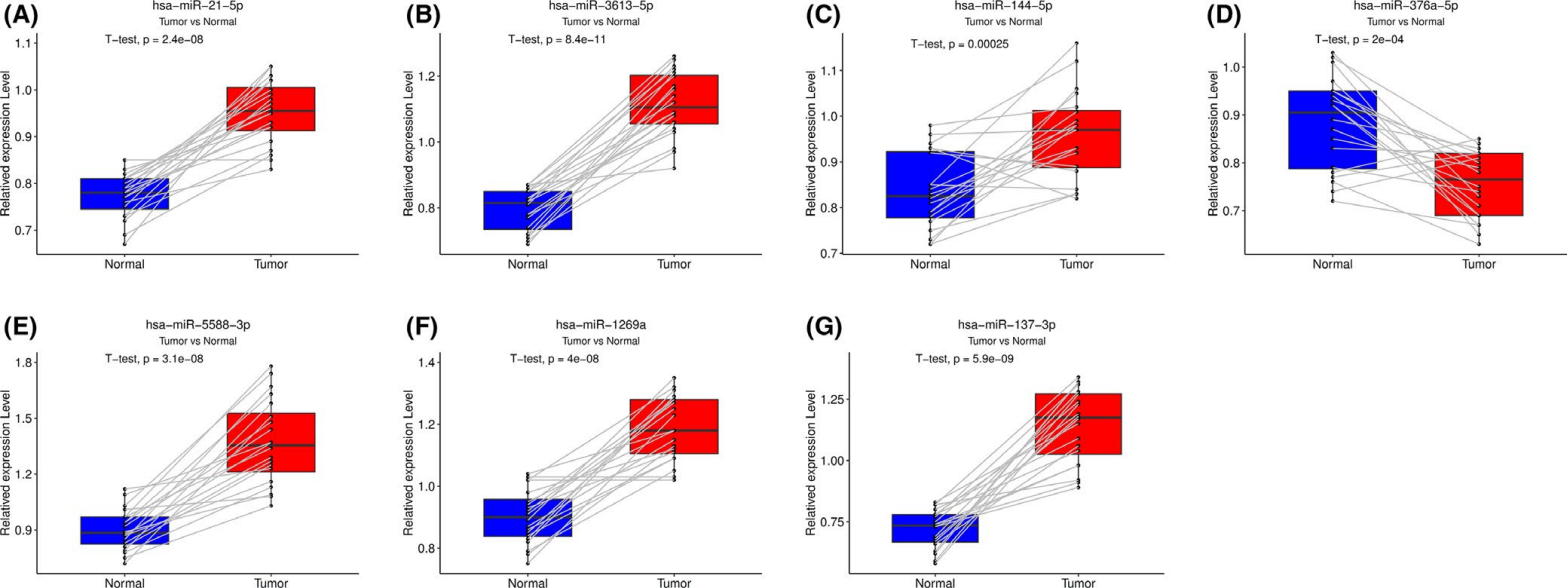
Experimental validation of 7 miRNAs between para‐tumor and KIRC tissue by qRT‐PCR. (A) hsamiR‐21‐5p, (B) hsa‐miR‐3613‐5p, (C) hsa‐miR‐144‐5p, (D) hsa‐miR‐376a‐5p, (E) hsa‐miR‐5588‐3p, (F) hsa‐miR‐1269a, and (G) hsa‐miR‐137‐3p

To verify the expressions of the seven miRNAs, qRT‐PCR was performed in para‐tumor tissues as well as in KIRC tissues. The results of qRT‐PCR revealed that six prognostic miRNAs were highly expressed in KIRC tissues while hsa‐miR‐376a‐5p was reduced (Figure 9).

## 4 DISCUSSION

Clear cell renal cell carcinoma (ccRCC) is a kind of solid tumor originating in renal tubules and insensitive to radiotherapy and chemotherapy.^15^, ^16^ It has been reported that the disorders of miRNAs may be involved in many diseases.^17^ With the development of technology, miRNAs have been demonstrated to be dysregulated in KIRC patients.^18^ Currently, there are few miRNAs‐clinical models for predicting the prognosis of KIRC. Herein, a new miRNAs‐clinical model was constructed and used to evaluate the prognosis of KIRC patients. We systemically explored the prognosis and function of significant miRNAs in KIRC. Clearly, 94 DEmiRNAs were identified in KIRC patients based on TCGA data. Subsequently, a 7‐miRNA signature was found and constructed, contributing to a better survival prediction in KIRC. Our findings may provide novel therapeutic targets for KIRC.

Hsa‐miR‐21‐5p, hsa‐miR‐3613‐5p, hsa‐miR‐144‐5p, hsa‐miR‐376a‐5p, hsa‐miR‐5588‐3p, hsa‐miR‐1269a, and hsa‐miR‐137‐3p were included in the 7‐miRNA model. MiR‐21 is an oncogenic miRNA that is upregulated in a variety of solid tumors. It has been reported that miR‐21‐5p promotes lung adenocarcinoma cell proliferation, migration, and invasion through targeting WWC2.^19^ Moreover, an increasing number of evidence has reported the involvement of miR‐21‐5p in KIRC prognosis.^20^, ^21^ MiR‐3613‐5p has been reported to be related to the metastasis of pancreatic cancer by targeting CDK6.^22^ Lu et al. have discovered that circRACGAP1 accelerates the progression of non‐small cell lung cancer by miR‐144‐5p/CDKL1 signaling.^23^ MiR‐1269a has been found to be associated with the occurrence and process of hepatocellular carcinoma by targeting oncogenes SPATS2L and LRP6.^24^ Previously, a 8‐miRNA KIRC model has been constructed by Qin et al.^25^ Interestingly, hsa‐miR‐3613‐5p, hsa‐miR‐144‐5p, and hsa‐miR‐1269a, which were incorporated in our model, were also found by them. MiR‐137‐3p, identified as a potential therapeutic target, suppresses the progression and metastasis of colorectal cancer cells by mediating the KDM1A‐dependent epithelial‐mesenchymal transition.^26^ The roles of miR‐376a‐5p and miR‐5588‐3p have not been studied in cancers until now. Currently, the biological roles of seven miRNAs have been seldom explored in KIRC. Therefore, the exact contributions of these miRNAs in KIRC need to be further investigated. In this study, a risk model with the 7 miRNAs was built. In this model, the Kaplan–Meier curve showed that the low‐risk group had a better survival rate. The ROC analysis revealed that this risk model contributed to the prediction of the prognosis of KIRC patients.

The GSEA results revealed many potential pathways for the miRNA model, among which adipocytokine signaling pathway, fatty acid metabolism, mTOR signaling pathway, PPAR signaling pathway, ErbB signaling pathway, and insulin signaling pathway were enriched in the low‐risk group. It is known that obesity has been considered an important dangerous factor for KIRC.^27^ Therefore, the adipocytokine signaling pathway and fatty acid metabolism may be involved in KIRC progression. Increasing evidence have shown that the mTOR signaling pathway,^28^ PPAR signaling pathway,^29^ ErbB signaling pathway,^30^ and insulin signaling pathway^31^ are significant cancer‐related pathways. Moreover, it has been found that DNA replication and P53 signaling pathway, highly correlated with the genesis and development of tumors, were enriched in high‐risk group.^32^, ^33^ Taken together, our results reveal the molecular regulatory network of the new model.

Next, the target genes of the seven miRNAs were predicted, which were performed in enrichment analysis. GO analysis indicated that the target genes were enriched in potassium ion transmembrane transport, potassium ion transport, and positive regulation of branching involved in ureteric bud morphogenesis, which was consistent with the previous studies in KIRC.^34^, ^35^ The results of KEGG revealed that the target genes were mainly enriched in transcriptional misregulation in cancer, Ras signaling pathway, Rap1 signaling pathway, PI3K‐Akt signaling pathway, Hippo signaling pathway, and calcium signaling pathway pathways. In fact, transcription regulation, Ras signaling pathway,^36^ Rap1 signaling pathway,^37^ PI3K‐Akt signaling pathway,^38^ Hippo signaling pathway,^39^, ^40^ and calcium signaling pathway pathways^41^ are involved in the occurrence and development of various cancers. Combined with these, our results not only suggest the association of the seven miRNAs with tumorigenesis and progression of KIRC, but also provide the potential pathways of these miRNAs in the treatment of KIRC.

The inclusion of clinical factors into the genetic model contributes to the improvement of the accuracy of the prediction model. In this study, a nomogram, which integrated the 7‐miRNA signature and two clinical independent risk factors to predict the prognosis of KIRC, was established. Notably, the miRNAs‐clinical nomogram had a good ability in predicting the prognosis of KIRC.

In fact, few prognostic miRNA models have been established in KIRC. In comparison with the previous models, more statistical methods were used to obtain prognostic miRNAs in our model. In addition, clinical features were integrated into this miRNA model in KIRC to build a nomogram, and this is the first report. Furthermore, the expressions of seven miRNAs were examined in KIRC tissues, which should be confirmed in the studies with larger sample sizes. In the future, more clinical databases should be used to verify the accuracy of the 7‐miRNA model.

In conclusion, we disclose a novel miRNA‐clinical prognosis model for KIRC, contributing to the evaluation of KIRC prognosis.

## CONFLICT OF INTEREST

The authors confirm that there are no conflicts of interest.

## ETHICS APPROVAL

The studies involving human participants were reviewed and approved by the Human Research Ethics Committee in The First Affiliated Hospital of Wenzhou Medical University. The patients/participants provided their written informed consent to participate in this study. Written informed consent was obtained from the individual(s) for the publication of any potentially identifiable images or data included in this article.

## Supporting information

Supplementary Material

## Data Availability

Publicly available datasets were analyzed in this study. This data can be found here: http://protal.gdc.cancer.gov, http://mirwalk.umm.uni‐heidelberg.de/, and https://david.ncifcrf.gov/.
