# Supplementary material for: A microRNA‐clinical prognosis model to predict the overall survival for kidney renal clear cell carcinoma
Source: Cancer Med. 2021 Jul 21;10(17):6128–39. doi: 10.1002/cam4.4148 (PMC8419758; doi:10.1002/cam4.4148)
Supplement: Supplementary file 1 — Supplementary Material [file CAM4-10-6128-s001.docx]

**Table S1**

24 prognostic related miRNAs selected by univariate Cox regression analysis.

| id | HR | HR.95L | HR.95H | P value |
| --- | --- | --- | --- | --- |
| hsa-miR-532-3p | 0.727809 | 0.602895 | 0.878605 | 0.000943 |
| hsa-miR-21-5p | 1.860427 | 1.509749 | 2.292558 | 5.69E-09 |
| hsa-miR-584-5p | 0.74661 | 0.615181 | 0.906118 | 0.003097 |
| hsa-miR-155-5p | 1.288993 | 1.14706 | 1.448488 | 2E-05 |
| hsa-miR-3613-5p | 1.635492 | 1.267078 | 2.111026 | 0.000158 |
| hsa-miR-660-5p | 0.775906 | 0.652638 | 0.922456 | 0.004048 |
| hsa-miR-188-3p | 0.816874 | 0.708537 | 0.941775 | 0.005331 |
| hsa-miR-142-3p | 1.237963 | 1.066 | 1.437668 | 0.005149 |
| hsa-miR-885-5p | 0.879395 | 0.813663 | 0.950436 | 0.001185 |
| hsa-miR-149-5p | 1.326866 | 1.170491 | 1.504132 | 9.85E-06 |
| hsa-miR-335-5p | 1.184177 | 1.051496 | 1.333601 | 0.005301 |
| hsa-miR-142-5p | 1.326798 | 1.131162 | 1.556269 | 0.000512 |
| hsa-miR-214-3p | 1.211625 | 1.062902 | 1.381159 | 0.004067 |
| hsa-miR-138-5p | 1.230839 | 1.109995 | 1.36484 | 8.18E-05 |
| hsa-miR-1251-5p | 0.863253 | 0.796295 | 0.93584 | 0.000357 |
| hsa-miR-204-5p | 0.890019 | 0.84162 | 0.941203 | 4.43E-05 |
| hsa-miR-144-5p | 0.777184 | 0.700796 | 0.861898 | 1.79E-06 |
| hsa-miR-144-3p | 0.829961 | 0.75018 | 0.918228 | 0.000301 |
| hsa-miR-376a-5p | 1.358658 | 1.146041 | 1.610721 | 0.000416 |
| hsa-miR-5588-3p | 0.77183 | 0.655362 | 0.908997 | 0.001914 |
| hsa-miR-1228-5p | 1.388705 | 1.163685 | 1.657238 | 0.000272 |
| hsa-miR-1269b | 1.068955 | 1.020239 | 1.119998 | 0.005081 |
| hsa-miR-1269a | 1.083628 | 1.046447 | 1.122129 | 6.52E-06 |
| hsa-miR-137-3p | 1.215544 | 1.094889 | 1.349494 | 0.000253 |


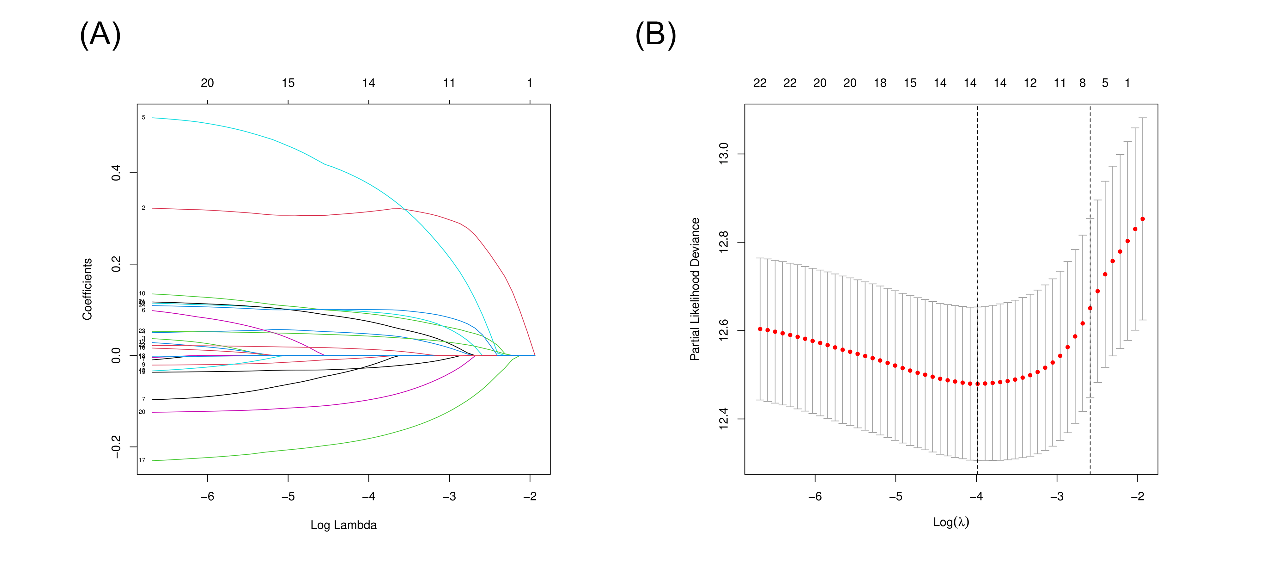


Figure S1 Lasso Cox regression analysis to select miRNAs. (A) Profile of the Lasso coefficient for each survival related miRNAs. (B) Plot of partial likelihood deviance. The corresponding λ value of the left broken line was considered as minimal λ value. 14 miRNAs were picked out according to the minimal λ.


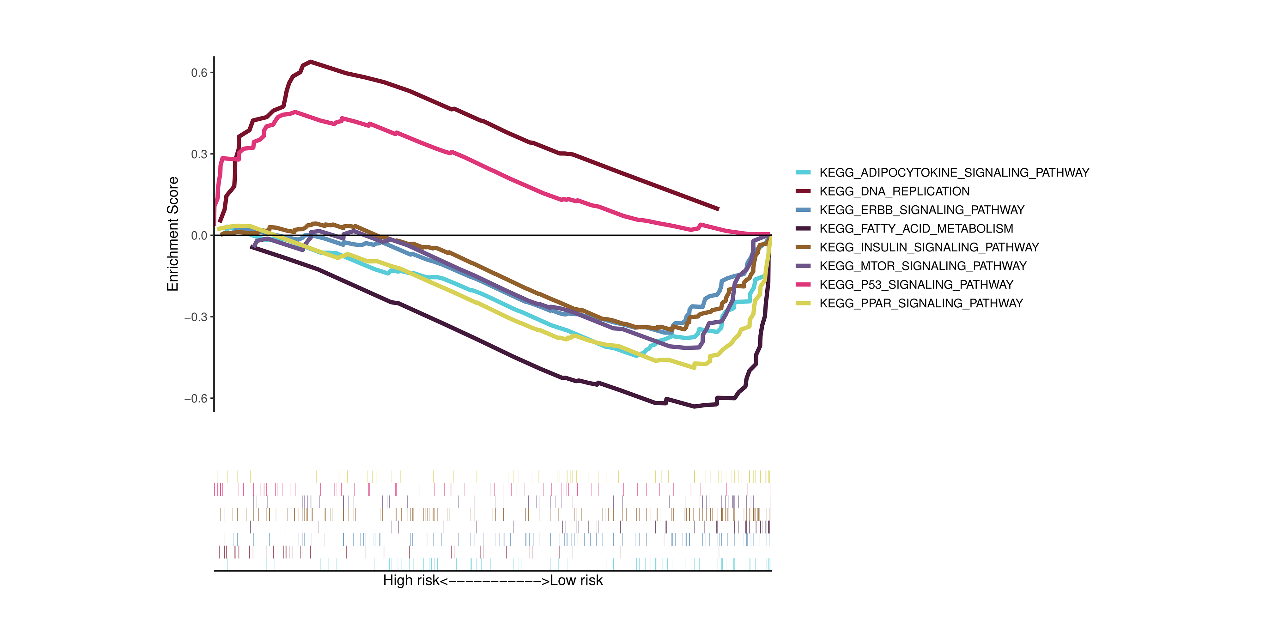


Figure S2 The GSEA enrichment result of crucial pathways between the high- and low-risk group.


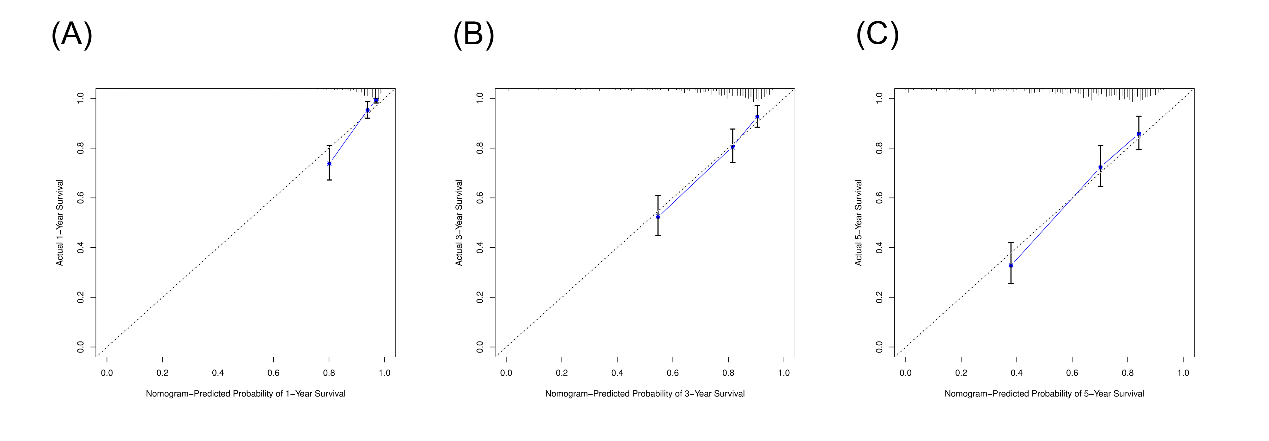


Figure S3 The calibration curves of the nomogram within 1-year (A), 3-year (B) and 5-year (C), respectively.
